# Immunostimulatory Effects of Chitooligosaccharides on RAW 264.7 Mouse Macrophages via Regulation of the MAPK and PI3K/Akt Signaling Pathways

**DOI:** 10.3390/md17010036

**Published:** 2019-01-08

**Authors:** Yue Yang, Ronge Xing, Song Liu, Yukun Qin, Kecheng Li, Huahua Yu, Pengcheng Li

**Affiliations:** 1 Key Laboratory of Experimental Marine Biology, Institute of Oceanology, Chinese Academy of Sciences, No. 7 Nanhai Road, Qingdao 266071, China; yy100462@163.com (Y.Y.); sliu@qdio.ac.cn (S.L.); ykqin@qdio.ac.cn (Y.Q.); lkc@qdio.ac.cn (K.L.); yuhuahua@qdio.ac.cn (H.Y.); 2 Laboratory for Marine Drugs and Bioproducts of Qingdao National Laboratory for Marine Science and Technology, No. 1 Wenhai Road, Qingdao 266237, China; 3 College of Earth and Planetary Sciences, University of Chinese Academy of Sciences, Beijing 100049, China; 4 Center for Ocean Mega-Science, Chinese Academy of Sciences, 7 Nanhai Road, Qingdao 266071, China

**Keywords:** chitooligosaccharide, immunostimulatory activity, RAW 264.7 cells, mitogen-activated protein kinases (MAPK), phosphoinositide 3-kinases (PI3K)/Akt

## Abstract

Chitooligosaccharides (COS), the hydrolyzed products of chitin and chitosan, can be obtained by various methods. In this study, water-soluble COS were prepared from α- and β-chitosan by microwave-assisted degradation and their immunostimulatory effects were investigated in RAW 264.7 macrophages. The results indicated that α-COS were more active than β-COS in promoting the production of nitric oxide (NO) and cytokines, such as tumor necrosis factor-α (TNF-α) and interleukin 6 (IL-6). Quantitative real-time reverse transcription polymerase chain reaction and Western blotting indicated that COS also enhanced the expression of inducible nitric oxide synthase (iNOS), cyclooxygenase-2 (COX-2), and TNF-α. Further analyses demonstrated that COS induced the phosphorylation of extracellular signal-regulated kinase (ERK), c-Jun N-terminal kinase (JNK), p38, p85 and Akt, and the nuclear translocation of p65, indicating that they are able to activate the mitogen-activated protein kinases (MAPKs) and phosphoinositide 3-kinases (PI3K)/Akt signaling pathways dependent on nuclear factor (NF)-κB activation. In conclusion, COS activate RAW 264.7 cells via the MAPK and PI3K/Akt signaling pathways and are potential novel immune potentiators.

## 1. Introduction

Immune responses are initiated by host defenses against invading pathogens via the innate and adaptive immune systems [1]. The immune responses of the elderly and patients with immunodeficiency diseases need to be enhanced. Therefore, immunoenhancing nutraceuticals and medicines have attracted a great deal of attention owing to their immunostimulatory activity. Macrophages and dendritic cells play pivotal roles in the immune system and activate immune responses via cytokine release, phagocytosis, and antigen presentation [2]. Macrophage activation is crucial for promoting immune activity [3]. Stimulated macrophages release various proinflammatory mediators and cytokines, including nitric oxide (NO), tumor necrosis factor-α (TNF-α), prostaglandin E_2_ (PGE_2_), interleukin-1β (IL-1β), and interleukin-6 (IL-6) [4,5]. These cytokines are secreted via activation of signaling pathways such as the mitogen-activated protein kinase (MAPK) and phosphoinositide 3-kinase (PI3K)/Akt, and via transcription factors like activator protein 1 (AP-1) and nuclear factor (NF)-κB.

Chitin is a naturally abundant substance exists in the fungal cell walls, exoskeletons of insects, and shells of crustaceans. An estimated 10^11^ tons of chitin are produced from living organisms annually [6]. Chitosan, a partially deacetylated product of chitin, has been widely studied and used in biomedical applications [7,8,9]. However, the pKa of chitosan is 6.5, and chitosan dissolves only under acidic conditions via the protonation of amino groups, which restricts its application. Nevertheless, chitosan is readily degraded into chitooligosaccharides (COS) by microwave irradiation, enzymes, or conventional heating. COS consist of 2–20 GlcN or GlcNAc units linked by β-1,4-*O*-glycoside bonds. The COS segments differ in the fraction of N-acetylated residues and the sequences of GlcN and GlcNAc residues [10].

COS possess many biological activities, including immunoregulatory, antiviral, anti-tumor, antibacterial, and antifungal activities [11]. Xing et al. [12] found that COS prepared by chemical hydrolysis, enzymatic hydrolysis, and microwave irradiation all improved innate and adaptive immunity, and COS degraded by microwave irradiation showed the finest activity. Through both in vitro and in vivo models, Zhang et al. [13] found that COS with a degree of polymerization (DP) of 3–8 promoted innate and adaptive immunity. Mei et al. [14], COS (DP 4–11) reported that COS (DP 4–11) exhibit strong immunostimulatory activity and protective effects in immunosuppressed mice. COS with chain lengths greater than six were more biologically active and had greater immunopotentiation activity than smaller COS [11].

Although some studies have reported on the immunoregulatory activity of COS, little is known of the molecular mechanisms involved. Therefore, it is not clear how COS exert their immunomodulatory activity. Feng et al. [15] demonstrated that COS promoted the release of IL-1β and TNF-α in macrophages. The promotion of cytokine gene transcription and protein expression by COS can affect immunity [16], but the detailed molecular mechanism of the immunomodulatory activity of COS is unclear, and the activity of β-COS has not been investigated. In this study, water-soluble α- and β-COS were obtained by microwave-assisted degradation. Then, the immunomodulatory activity of COS was investigated in RAW 264.7 cells, and the COS-induced signaling pathways were characterized to elucidate the molecular mechanisms.

## 2. Results

### 2.1. Characterization of COS

**Figure 1:**
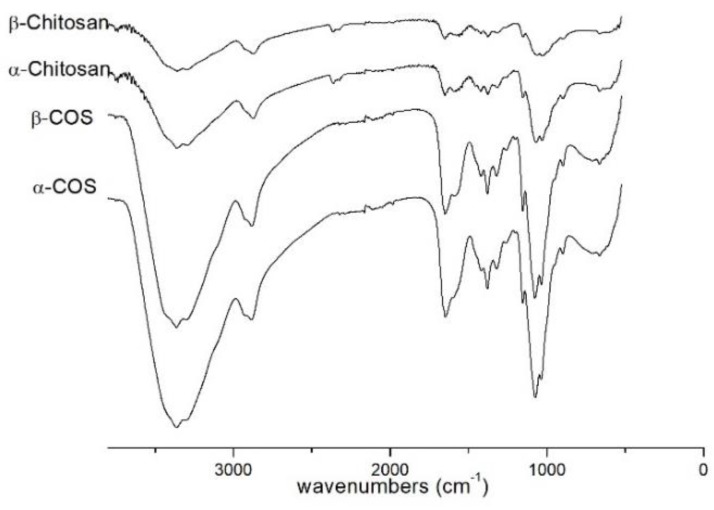
Fourier-transform infrared (FT-IR) spectra of chitosan and chitooligosaccharide (COS). The molecular weights were as follows: α-chitosan (1856 kDa), β-chitosan (4574 kDa), α-COS (1874 Da) and β-COS (2186 Da).

The IR spectra of α-chitosan (1856 kDa), β-chitosan (4574 kDa), α-COS (1874 Da), β-COS (2186 Da) were shown in Figure 1. The characteristic absorption peaks appeared at 1651 cm^−1^ (Amide I), 1591 cm^−1^ (N-H bending) and 1375 cm^−1^ (Amide III). The spectrum of α-COS and β-COS were similar to α- and β-chitosan with high molecular weight.

**Figure 2:**
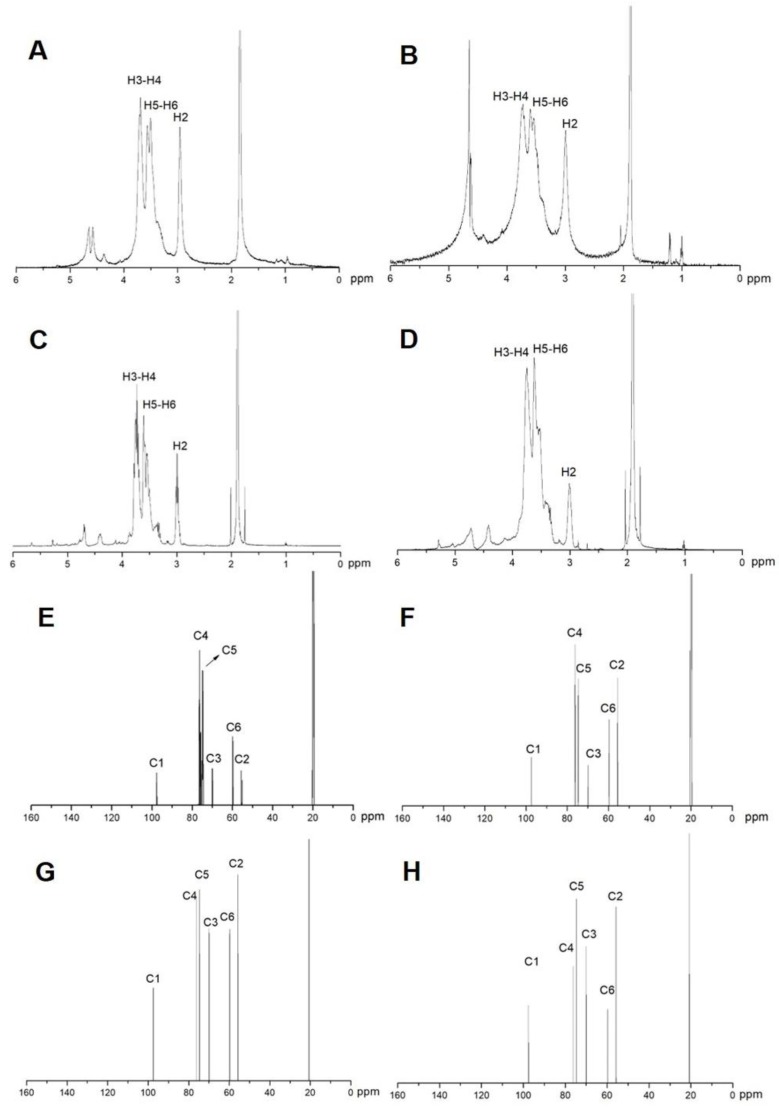
^1^H NMR and ^13^C NMR spectra of chitosan and chitooligosaccharide (COS). (**A**) ^1^H NMR spectrum of α-chitosan (1856 kDa). (**B**) ^1^H NMR spectrum of β-chitosan (4574 kDa). (**C**) ^1^H NMR spectrum of α-COS (1874 Da). (**D**) ^1^H NMR spectrum of β-COS (2186 Da). (**E**) ^13^C NMR spectrum of α-chitosan (1856 kDa). (**F**) ^13^C NMR spectrum of β-chitosan (4574 kDa). (**G**) ^13^C NMR spectrum of α-COS (1874 Da). (**H**) ^13^C NMR spectrum of β-COS (2186 Da).

The ^1^H NMR and ^13^C NMR spectrum of α-chitosan (1856 kDa), β-chitosan (4574 kDa), α-COS (1874 Da) and β-COS (2186 Da) were shown in Figure 2. As shown in Figure 2C, the signal of α-COS (1874 Da) at 3.00 ppm was attributed to H2, the signals between 3.52 and 3.62 ppm were assigned to H5 and H6, the peaks at 3.75 ppm were attributed to H3 and H4. The peaks were similar to α-chitosan (1856 kDa) as shown in Figure 2A. The ^13^C NMR spectrum of α-COS (1887 Da) was plotted in Figure 2G. The signals at 97.48, 55.67, 69.98, 76.18, 74.67, 59.80 were attributed to C1, C2, C3, C4, C5 and C6, respectively. These signals were also similar to those of the 1856-kDa chitosan (Figure 2E). As shown in Figure 2D,H, the ^1^H NMR and ^13^C NMR spectrum of β-COS (2186 Da) were plotted, and the signals were assigned. The signals of β-chitosan (4574 kDa) were identical with those of β-COS (2186 Da). Moreover, apart from a slight difference in signal intensity, the signals of β-chitosan and β-COS were also identical with those of α-chitosan and α-COS.

The difference of α- and β-COS originates from the antiparallel configuration of α-COS and the parallel structure of β-COS. The structure unit of COS was not destructed by microwave degradation demonstrated by the IR, ^1^H NMR, and ^13^C NMR spectra.

### 2.2. NO Production

**Figure 3:**
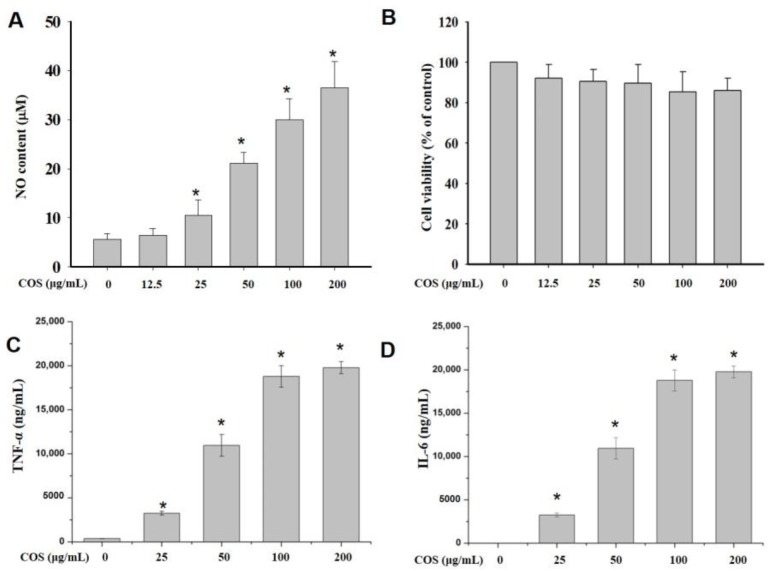
The effects of chitooligosaccharides (COS) on cell viability and cytokine production. RAW 264.7 cells were treated with COS (0–200 μg/mL) for 24 h. (**A**) The nitric oxide production on treatment with α-COS (1874 Da). (**B**) The cell viability on treatment with α-COS (1874 Da). (**C**) The production of tumor necrosis factor-α (TNF-α) on treatment with α-COS (1874 Da). (**D**) The production of interleukin-6 (IL-6) on treatment with α-COS (1874 Da). The values are presented as means ± SD (n = 3). (* *p* < 0.05 vs. control group).

To elucidate the immunological effect of COS, the cells were pretreated with various concentrations and configurations of COS, and NO production was measured using Griess reagent. As shown in Figure S1, the NO-promoting activity of α-COS was better than that of β-COS. The most effective COS had a molecular weight of 1874 Da (COS 1874 Da) and was chosen for further investigation. The results indicated that the production of NO by macrophages was enhanced by increasing the COS concentration from 12.5 to 200 μg/mL (Figure 3A). Furthermore, to eliminate lipopolysaccharide contamination, a ToxinSensor^TM^ Chromogenic LAL Endotoxin Assay Kit (GenScript, Piscatway, NJ, USA) was used to perform an endotoxin assay. The results showed that the samples were not contaminated by endotoxin.

### 2.3. Cell Viability

The cytocompatibility of different concentrations of COS in RAW 264.7 cells was evaluated by the MTT assay, and the blank control group was designated as 100% cell viability. The results showed that both α- and β-COS (100 μg/mL) had good biocompatibility after the pretreatment of RAW 264.7 cells for 24 h (Figure S2).

Figure 3B displays the effects of various concentrations of COS 1874 Da on macrophage viability. The cell viability with 12.5‒200 μg/mL COS 1874 Da exceeded 80%, indicating that COS 1874 Da had no obvious toxicity in RAW 264.7 cells.

### 2.4. COS Induced the Production of Cytokines in Macrophages

**Figure 4:**
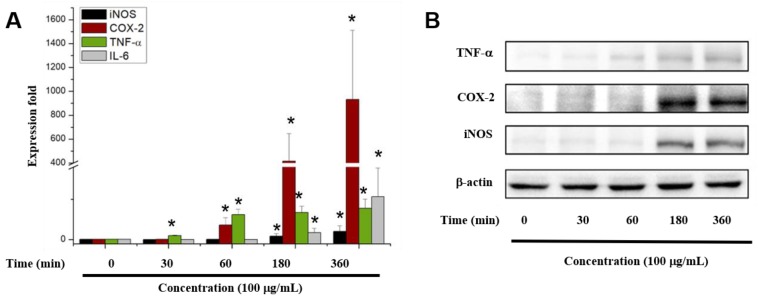
Effects of COS on the expression of cytokines in RAW 264.7 cells at the gene and protein levels. (**A**) Gene expression of inducible nitric oxide synthase (iNOS), TNF-α, IL-6, and cyclooxygenase (COX)-2 in RAW264.7 cells determined by real-time reverse transcription polymerase chain reaction after stimulation with 100 μg/mL COS (1874 Da). The expression data were normalized to glyceraldehyde 3-phosphate dehydrogenase and shown as fold-changes relative to the control (presented as means ± SD) (* *p* < 0.05 vs. control group). (**B**) The effects of 100 μg/mL COS (1874 Da) on the protein expression levels of TNF-α, iNOS, and COX-2.

To determine cytokine expression levels after COS treatment, cytometric bead array (CBA) experiments were conducted. Figure 4C,D shows that the production of TNF-α and IL-6 was obviously promoted by COS treatment (25, 50, 100 and 200 μg/mL) in a dose-dependent manner. The cytokine production was increased to nearly 20,000 ng/mL at a concentration of 200 μg/mL.

### 2.5. COS Enhanced Cytokine Gene and Protein Expression

To investigate whether COS can regulate the levels of cytokine transcription, RAW 264.7 cells were pretreated with 100 μg/mL COS 1874 Da for 0, 0.5, 1, 3 and 6 h. Then, the iNOS, COX-2, IL-6, and TNF-α gene levels were detected by real-time RT-PCR. Quantitative real-time PCR (Figure 4A) indicated that the expression of cytokine genes after a 360 min COS treatment was increased more than 20-fold compared with untreated cells, and the expression of COX-2 reached ca. 1000-fold. To validate these results, immunoblotting assays were performed. As shown in Figure 4B, the expression of TNF-α, IL-6, and COX-2 was obviously promoted after 180 min. These results confirmed the effect of COS on promoting inflammatory mediators in RAW 264.7 macrophages.

### 2.6. COS Interferes with the NF-κB, MAPK and PI3K/Akt Signaling Pathways

**Figure 5:**
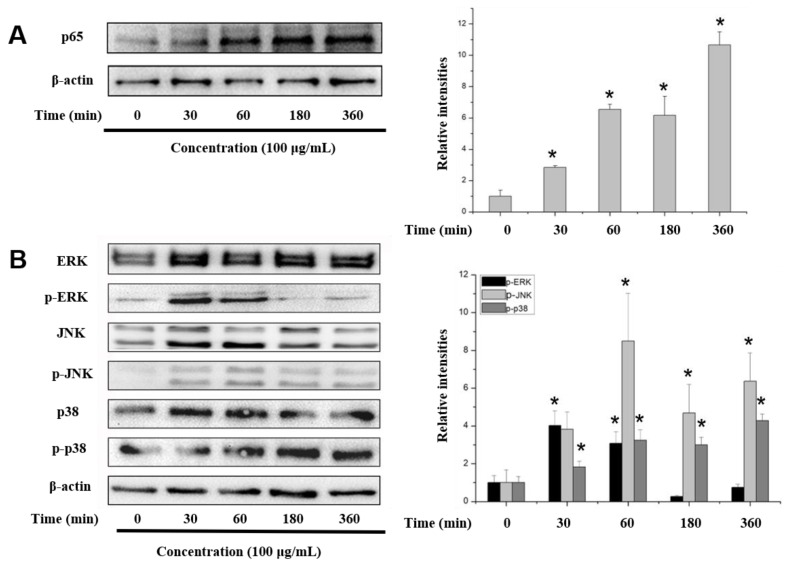

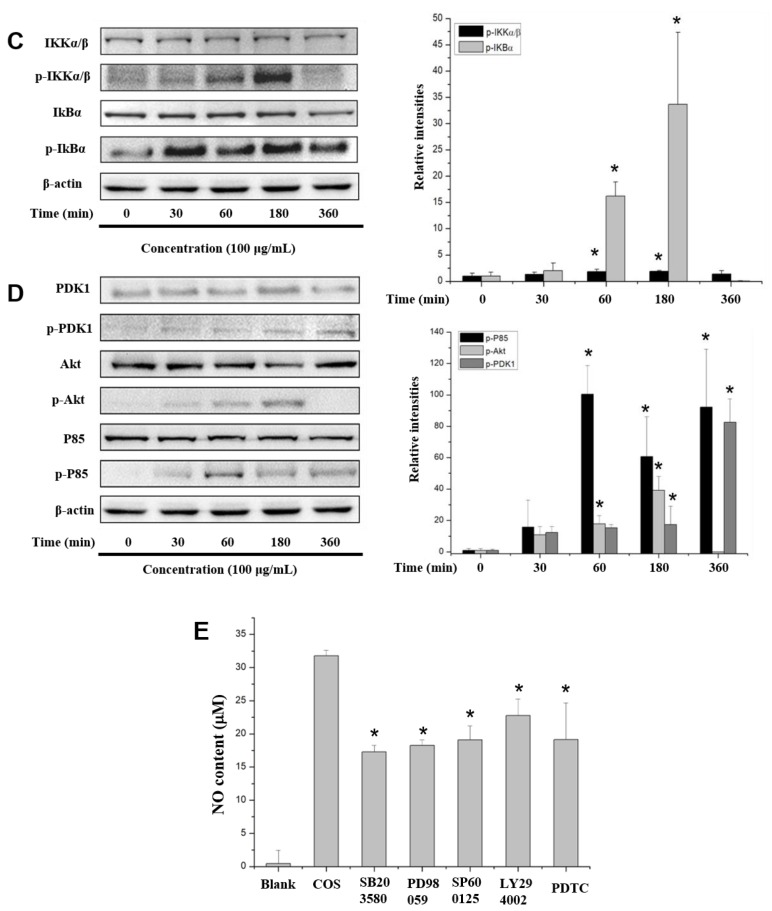
Determination of COS-induced signaling pathways. RAW 264.7 cells were stimulated with 100 μg/mL COS (1874 Da) for the indicated times (0–360 min). Then, cell proteins were extracted and subjected to Western blotting. The effects of COS (1874 Da) on the NF-κB p65 (**A**), MAPK (**B**), IKKα/β and IκBα (**C**), PI3K-Akt (**D**) signaling pathways were determined. The relative expression levels of protein were normalized by β-actin. (**E**) NO production in RAW 264.7 cells pretreated with or without inhibitors after stimulated with 100 μg/mL COS. Data are presented as mean ± SD. (* *p* < 0.05 vs. COS-treated group).

Immunoblotting was used to examine the possible involvement of NF-κB in the induction of proinflammatory mediators by COS. As shown in Figure 5A, the expression of p65, a key subunit of NF-κB, increased in a time-dependent manner after COS treatment. Furthermore, 100 μg/mL COS promoted the phosphorylation of IκBα and the upstream phosphorylation of IKKα/β complex (the upstream kinase of IκB) protein (Figure 5C). These results indicated that COS promotes IκBα degradation and activated the translocation of NF-κB.

To investigate the participation of the MAPK signaling pathway, cells were treated with COS for 0, 30, 60, 180 and 360 min, and then the phosphorylation of c-Jun N-terminal kinase (JNK), extracellular signal-regulated kinase (ERK), and p38 proteins was determined. The phosphorylation of ERK was obviously promoted at 30 min but decreased after 180 min (Figure 5B). The expression of p-JNK was promoted after treatment for 30 to 360 min. The phosphorylation of p38 was significantly promoted and reached a plateau after 180 min.

The phosphorylation of the members of the PI3K-Akt signaling pathway after COS treatment was also investigated. As shown in Figure 5D, the phosphorylated Akt levels peaked at 180 min. COS also promoted the phosphorylation of PDK1 in a time-dependent manner. Moreover, COS also stimulated p85 protein, the regulatory subunit of PI3K, which peaked at 60 min (Figure 5D).

To validate the involvement of MAPK, PI3K/Akt and NF-κB pathways in COS-induced NO production, the effects of specific inhibitors on NO production were determined in RAW 264.7 cells. As shown in Figure 5E, all the inhibitors suppressed the production of NO in COS-induced RAW 264.7 cells, further demonstrating that COS promote NO production by activating the MAPK, PI3K/Akt and NF-κB pathways.

## 3. Discussion

NO, a small diffusible molecule, is a short-lived, endogenously produced gas synthesized mainly by iNOS [17]. As an effector molecule, NO may be responsible for eradicating invaded pathogens in macrophages [18]. Therefore, we determined the NO expression in RAW 264.7 cells treated with 100 μg/mL α- and β-COS. The results indicated that α-COS is more active at promoting NO production than β-COS. Then, we explored the molecular mechanisms of α-COS. After pretreatment with various concentrations of COS, the NO production in RAW 264.7 cells increased in a dose-dependent manner. Besides NO, pro-inflammatory cytokines are also pivotal in the destruction of pathogens. COS increased the production of two cytokines, TNF-α and IL-6, as determined by CBA and confirmed by RT-PCR and immunoblotting. Our results were consistent with the report by Wu et al. [19], who found that low-molecular-weight chitosan (3 and 50 kDa) considerably induced the expression of IL-6, TNF-α, NO, interferon-γ, and iNOS in a molecular weight- and concentration-dependent manner. Moreover, Wei et al. [16] also found that (GLcN)_5_ and (GLcN)_6_ promoted the gene expression and protein secretion of TNF-α, IL-1, and IFN-γ.

To explore the mechanisms of macrophage activation, we performed Western blot analysis of proteins involved in the NF-κB signaling pathway. The transcription factor NF-κB play vital roles in the innate and adaptive immune systems. NF-κB is related to the transcription of many mediators and proinflammatory cytokines genes, such as iNOS, IL-1β, TNF-α, and COX-2 [20,21,22]. As we demonstrated (Figure 5A), the subunit of NF-κB (p65) was phosphorylated on stimulation with COS. To explore the upstream pathway, the phosphorylation of IκB kinase enzyme complex (IKK α/β) and IκBα proteins were determined. The results showed that COS activated the IKK enzyme complex, which phosphorylated the downstream IκBα protein. Then, IκB proteins were ubiquitylated and degraded, which allowed NF-κB to translocate to the nucleus and promoted the production of its target genes. Our results were consistent with Zheng et al. [23], who found that low-molecular-weight chitosans exert immunostimulatory activity via activation of the AP-1 and NF-κB pathways and 3 kDa chitosan showed greater activity than 5 kDa chitosan in RAW264.7 macrophages. Li et al. [24] also found that five chitooligomers, ranging from dimers to hexamers, promoted the expression of NF-κB downstream genes. Our results demonstrated that besides COS of 3 kDa and COS below 1 kDa, COS of 2 kDa could also promote the translocation of NF-κB.

MAPK activates the transcription of several transcription factors, including NF-κB and AP-1 [25]. Based on the finding that COS promoted NF-κB activation, the phosphorylation levels of ERK, JNK, and p38 proteins were determined. This showed that COS activated the MAPK signaling pathway during macrophage activation. As shown in Figure 5C, COS promoted the phosphorylation of Akt and the p85 subunit. Furthermore, the upstream PDK1 was also increasingly phosphorylated by COS in RAW 264.7 cells. Therefore, the PI3K/Akt pathway, another important pathway in regulating immunity responses, also participated in the activation of RAW 264.7 macrophages induced by COS.

However, it has been reported that COS exerts an anti-inflammatory effect in macrophages by inhibiting the phosphorylation of MAPKs, NF-κB and AP-1 [26]. Kim et al. [27] reported that after oral intake of COS, the IL-12 and interferon-γ levels of elderly people were increased, while the production of IL-1β and TNF-α decreased; the results suggested that COS (3500 Da) has dual immunostimulatory and anti-inflammatory effects in the elderly. These findings are distinct from our results. The different biological activities of COS may result from the differences in molecular weight, degree of DP, N-acetylation, arrangement of acetyl groups, fraction of N-acetylated residues, and pattern of N-acetylation [28].

COS exert their immune-enhancing effect by the interaction with receptors on the macrophage membrane surface, such as Toll-like receptor 4 (TLR 4) [13], complement receptor 3 [29] and mannose receptor [30]. The COS we used in this study is a complex mixture, so it is not clear which component led to the immunostimulatory effect. Therefore, we need to further explore the receptors binding with COS. A remaining challenge is to identify the most active molecules using a component with a single DP.

## 4. Materials and Methods

### 4.1. Chemicals and Reagents

α-Chitosan with an average molecular weight of 1856 kDa and degree of deacetylation of 86.0% was provided by Qingdao Yunzhou Biochemical Corp. (Qingdao, China). *Loligo japonica* squid pens were obtained from Yongming Food Co., Ltd. (Liaoning, China). LPS (*Escherichia coli* 0111: B4), 1-(4,5-dimethylthiazol-2-yl)-3,5-diphenyltetrazolium bromide (MTT), sulfanilamide and naphthylethylenediamine dihydrochloride were obtained from Sigma (St. Louis, MO, USA). The Roswell Park Memorial Institute (RPMI) medium 1640 and Penicillin-Streptomycin were purchased from Gibco BRL (Life Technologies, Shanghai, China). The fetal bovine serum (FBS) was obtained from HyClone (Thermo Fisher Scientific, Logan, UT, USA). The kits for cDNA synthesis were purchased from Invitrogen (Carlsbad, CA, USA). The SYBR Premix Ex Taq kits were obtained from Takara Bio (Dalian, China). Primary antibodies to TNF-α, cyclooxygenase (COX)-2, p85, PDK1, Akt, IκBα, p65, c-Jun N-terminal kinase (JNK), extracellular signal-regulated kinase (ERK), p38, and phospho-specific antibodies to p85, PDK1, Akt, IκBα, p65, ERK, JNK, p38 and β-actin were purchased from Cell Signaling Technology (Beverly, MA, USA) except the primary antibody against inducible NO synthase (iNOS) from Abcam (Cambridge, MA, USA). Horseradish peroxidase (HRP)-conjugated goat anti-mouse, and goat anti-rabbit IgG antibodies were purchased from Abcam (Cambridge, MA, USA). The mouse inflammation cytometric bead array (CBA) kit was provided by BD Biosciences (San Diego, CA, USA). Inhibitors including SB203580 (a p38 inhibitor), PD98059 (an ERK inhibitor), SP600125 (a JNK inhibitor) and PDTC (a NF-κB inhibitor) were purchased from Selleck (Shanghai, China). LY294002 (a PI3K inhibitor) was provided by Calbiochem (San Diego, CA, USA).

### 4.2. Preparation of α- and β-Chitooligosaccharide

The preparation of β-chitosan from Loligo Japonica squid pens was followed by previously described [31]. The preparation of COS was performed by microwave irradiation method. Briefly, chitosan powder (5 g) was dissolved in 250 mL 2% acetic acid, then H_2_O_2_ (5 mL) was added before being heated in laboratory microwave reaction equipment with an infrared reaction thermometer under magnetic stirring. After the reaction, the average molecular weights of the resulting products were measured by gel permeation chromatography (GPC) analysis. The mixture was neutralized by NaOH and then dialyzed with 500 Da cut-off dialysis membrane.

### 4.3. Characterization of Chitosan and Chitooligosaccharide

The average molecular weight of COS was measured by GPC analysis performed on TSK 3000-PWXL column (Tosoh, Tokyo, Japan) eluted with the mobile phases (0.1 M CH3COONa and 0.2 M CH3COOH aqueous solution). Fourier transform infrared (FT-IR) spectra of chitosan and chitooligosaccharide were performed by a Thermo Scientific Nicolet iS10 FT-IR spectrometer. The NMR spectra (^1^H and ^13^C) were documented by a JEOL JNM-ECP600 spectrometer (JEOL, Tokyo, Japan).

### 4.4. Cell Culture

The RAW 264.7 cell line was provided by the American Type Culture Collection (Manassas, VA, USA). Cells were maintained in RPMI 1640 medium containing 10% FBS (inactivated by heating at 56 °C for 30 min), l-glutamine (2 mM) and 1% penicillin-streptomycin in a humidified atmosphere with 5% CO_2_ at 37 °C.

### 4.5. Cell Proliferation Assay

RAW 264.7 macrophages were seeded onto 96-well flat-bottom culture plates (1 × 10^5^ cells/well) and allowed to adhere overnight. Thereafter, fresh medium containing indicated contents of chitooligosaccharide (0–200 µg/mL) were introduced and incubated for 24 h. Then the supernatants were discarded, and cells were cultured with 100 µL MTT solution (0.5 mg/mL) for 4 h, and the formed formazan crystals were dissolved in 100 µL MTT stopping buffer. After overnight cultivation, the absorbance was determined at 550 nm using a microplate reader (Tecan, Männedorf, Switzerland).

### 4.6. NO and Cytokines Quantitation

Followed the treatment as described in Section 2.5, the culture supernatants were collected for determining NO and cytokines production. The NO levels were determined as nitrite NO_2_^–^ by Griess reaction as previously reported [32]. The IL-6 and TNF-α levels were measured by C6 Plus flow cytometer (BD Biosciences, Sparks, MD, USA) using mouse inflammation cytometric bead array kits (San Diego, CA, USA).

### 4.7. The mRNA Expression Levels of Cytokines Determined by Reverse Transcription-Polymerase Chain Reaction

RAW 264.7 cells were treated by COS for different time periods (0, 30, 60, 180, 360 min). Then the total cellular RNA was extracted from various groups by Trizol^®^ reagent (Invitrogen, Carlsbad, CA, USA). The concentrations of RNA were detected by Nanodrop spectrophotometer (NanoDrop Technologies, Wilmington, DE, USA) followed by cDNA synthesis. Then quantitative real-time polymerase chain reaction (PCR) was performed. The nucleotide sequences primers are shown in Table S1. QRT-PCR was carried out, and the fold increase of each gene was calculated using the 2^−ΔΔCT^ method [33]. Housekeeping gene glyceraldehyde 3-phosphate dehydrogenase (GAPDH) was set as the internal reference.

### 4.8. Immunoblotting 

For immunoblot analysis, RAW 264.7 macrophages were seeded in 6-well culture plates (5 × 10^5^ cells/well) and cultured with COS for different time periods (0, 30, 60, 180, 360 min). Cells were scraped off, then the cytoplasmic and nuclear proteins were extracted. Briefly, cells were collected and lysed by 200 μL buffer (ComWin Biotech, Beijing, China). Then the total proteins were extracted by a Roche Complete protease inhibitor cocktail (Roche Diagnostics Ltd., Mannheim, Germany) and collected by centrifugation. Nuclear proteins were extracted using nuclear protein isolation kits (ComWin Biotech, Beijing, China) according to the manufacturer’s instructions. The protein concentration was determined by BCA protein assay kits (Com Win Biotech). Then equal grams of proteins (40 μg) were loaded and separated by sodium dodecyl sulfate (SDS)-polyacrylamide gel electrophoresis (PAGE) and then transferred to a polyvinylidene fluoride (PVDF) membrane. After blocked by BSA and rinsed by TTBS, the membranes were incubated with primary and HRP-labeled antibodies. The blot was imaged by Tanon-5200 chemiluminescence detection system (Tanon Science, Shanghai, China). The band density was quantified by Quantity One software (Bio-Rad, Munich, Germany).

### 4.9. Statistical Analysis

Data were presented as mean ± SD, the significance among different groups was analyzed by one-way analysis of variance. *p*-values < 0.05 were regarded as statistically significant. 

## 5. Conclusions

In conclusion, the NO production of macrophages pretreated with α- and β-COS was compared. The results suggested that α-COS is more active than β-COS. Therefore, α-COS was chosen for further study. The CBA, RT-PCR, and Western blotting results demonstrated that COS had immunostimulatory effects in RAW 264.7 cells. It promoted the secretion of NO and proinflammatory cytokines, including TNF-α, IL-6, and COX-2, via the PI3K-Akt and MAPK pathways and NF-κB activation. COS possesses great potential as a novel candidate for the treatment of immunosuppressive diseases and as a vaccine adjuvant.

## Supplementary Materials

The following are available online at http://www.mdpi.com/1660-3397/17/1/36/s1, Figure S1: Nitric oxide production treated with α-chitooligosaccharide (α-COS) and β- chitooligosaccharide (β-COS) at the concentration of 100 μg/mL., Figure S2: The cell viability treated with α-COS and β-COS at the concentration of 100 μg/mL. Table S1: The primer sequences and conditions for RT-PCR.
