# Supplementary material for: Immunostimulatory Effects of Chitooligosaccharides on RAW 264.7 Mouse Macrophages via Regulation of the MAPK and PI3K/Akt Signaling Pathways
Source: Mar Drugs. 2019 Jan 8;17(1):36. doi: 10.3390/md17010036 (PMC6357175; doi:10.3390/md17010036)
Supplement: Supplementary file 1 [file marinedrugs-17-00036-s001.pdf]

Immunostimulatory Effects of Chitooligosaccharides on RAW 264.7 Mouse Macrophages via Regulation of the MAPK and PI3K/Akt Signaling Pathways

Yue Yang [1,2,3,4], Ronge Xing [1,2,4,*], Song Liu [1,2,4], Yukun Qin [1,2,4], Kecheng Li [1,2,4], Huahua Yu [1,2,4] and Pengcheng Li [1,2,4,*]

[1] Key Laboratory of Experimental Marine Biology, Institute of Oceanology, Chinese Academy of Sciences, No. 7 Nanhai Road, Qingdao 266071, China; yy100462@163.com (Y.Y.); sliu@qdio.ac.cn (S.L.); ykqin@qdio.ac.cn (Y.Q.); lkc@qdio.ac.cn (K.L.); yuhuahua@qdio.ac.cn (H.Y.)

[2] Laboratory for Marine Drugs and Bioproducts of Qingdao National Laboratory for Marine Science and Technology, No. 1 Wenhai Road, Qingdao 266237, China

[3] College of earth and planetary sciences, University of Chinese Academy of Sciences, Beijing 100049, China

[4] Center for Ocean Mega-Science, Chinese Academy of Sciences, 7 Nanhai Road, Qingdao 266071, China

* Correspondence: xingronge@qdio.ac.cn (R.X.); pcli@qdio.ac.cn (P.L.); Tel.: +86-532-8289-8707 (P.L.); Fax: +86-532-8296-8780 (R.X.)

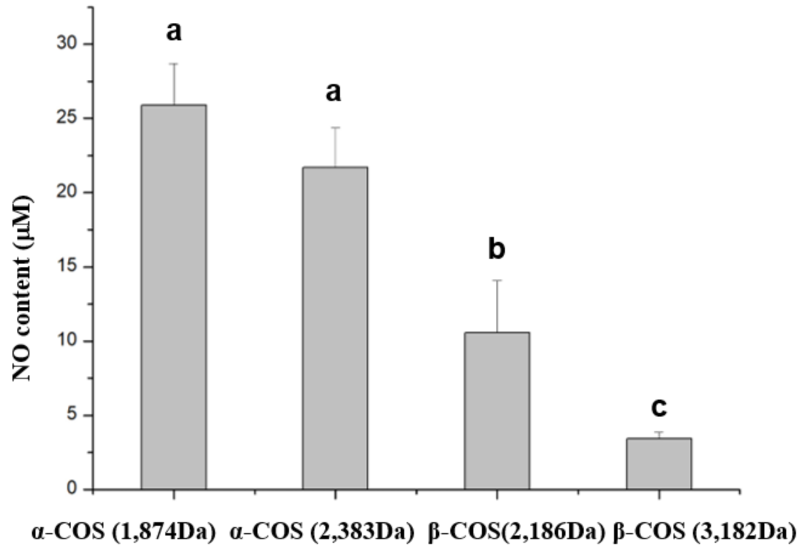

Figure S1. Nitric oxide production treated with α-chitooligosaccharide (α-COS) and β-chitooligosaccharide (β-COS) at the concentration of 100 μg/mL. The values are presented as means ± SD (n = 3). Values with the same superscript letters were not significantly different from each other at $p < 0.05$.

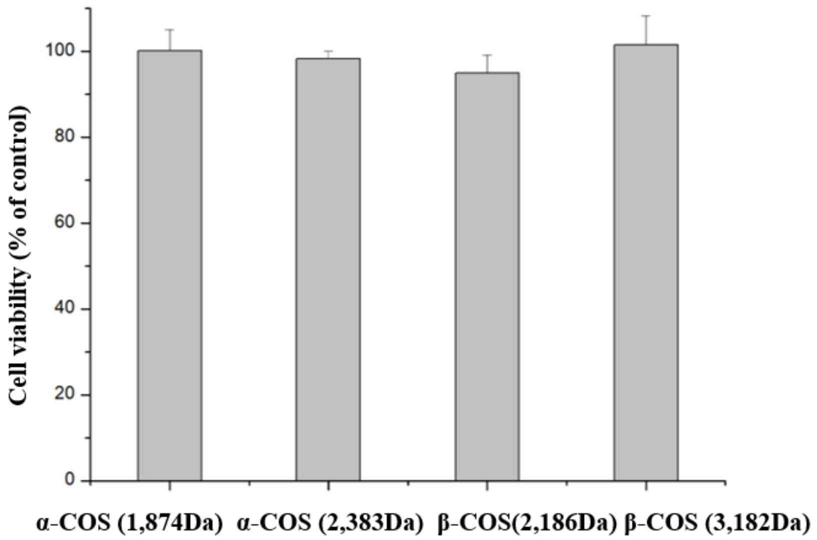

Figure S2. The cell viability treated with α-COS and β-COS at the concentration of 100 μg/mL. The values are presented as means ± SD (n = 3).

Table 1 The primer sequences and conditions for RT-PCR

Genes	Primer Sequence（5′–3′）	Denaturation	Annealing	Extension	Cycles
GAPDH	F: ACTCACGGCAAATTCAACGGCA<br>R: GACTCCACGACATACTCAGCAC		60 °C, 30 s		25
iNOS	F: CCCTTCCGAAGTTTCTGGCAGCAG<br>R: GGCTGTCAGAGCCTCGTGGCTTTGG		55 °C, 30 s		25
COX-2	F: CACTACATCCTGACCCACTT<br>R: ATGCTCCTGCTTGAGTATGT	94 °C,30 s	55 °C, 30 s	72 °C, 30 s	25
TNF-α	F: TGCCTATGTCTCAGCCTCTTC<br>R: GAGGCCATTTGGGAACTTCT		55 °C, 30 s		25
IL-6	F: AGACTTCCATCCAGTTGCCTTCTTG<br>R: CATGTGTAATTAAGCCTCCGACTTGTG		55 °C, 30 s		25
